# Risk of cancer among individuals with a history of bacterial sexually transmitted infections: A population‐based study in Alberta, Canada

**DOI:** 10.1002/ijc.70215

**Published:** 2025-11-14

**Authors:** Hina M. Qureshi, Taylor Hughes, Eduardo L. Franco, Kirsten M. Fiest, Jennifer Gratrix, Petra A. Smyczek, Ronald Read, Arfan R. Afzal, Rob Deardon, Aliya Kassam, Miranda M. Fidler‐Benaoudia

**Affiliations:** ^1^ Department of Community Health Sciences University of Calgary Calgary Alberta Canada; ^2^ Department of Cancer Epidemiology and Prevention Research Cancer Care Alberta Calgary Alberta Canada; ^3^ Division of Cancer Epidemiology McGill University Montreal Quebec Canada; ^4^ Department of Critical Care Medicine, Cumming School of Medicine University of Calgary Calgary Alberta Canada; ^5^ Department of Psychiatry, Cumming School of Medicine University of Calgary Calgary Alberta Canada; ^6^ Sexually Transmitted Infections Services Primary Care Alberta Edmonton Alberta Canada; ^7^ Faculty of Medicine and Dentistry University of Alberta Edmonton Alberta Canada; ^8^ Faculty of Medicine University of Calgary Calgary Alberta Canada; ^9^ Faculty of Nursing University of Calgary Calgary Alberta Canada; ^10^ Faculty of Veterinary Medicine University of Calgary Calgary Alberta Canada; ^11^ Department of Mathematics and Statistics, Faculty of Science University of Calgary Calgary Alberta Canada; ^12^ Department of Oncology, Cumming School of Medicine University of Calgary Calgary Alberta Canada

**Keywords:** cancer causes, chlamydia, gonorrhea, sexually transmitted infections, syphilis

## Abstract

We investigated cancer risk among individuals with a prior bacterial sexually transmitted infection (STI) diagnosis using a retrospective cohort study of all Albertan residents diagnosed with chlamydia, gonorrhea, or syphilis during 2000–2014, that was then linked to the Alberta Cancer Registry. Follow‐up started 5 years from their first bacterial STI diagnosis and continued until the first instance of a cancer diagnosis, death, or the study end date (December 31, 2019). Internal comparisons between the bacterial STI groups were undertaken using adjusted hazard ratios, while sex‐specific standardized incidence ratios (SIRs) were calculated to compare cancer risk with the Alberta general population. The likelihood of developing cancer was largely comparable within the bacterial STI cohort, though head/neck cancer was more common after only gonorrhea exposure, and lung cancer was more common after only syphilis exposure. When compared with the general population, statistically significant SIRs were observed among females for cervical cancer (SIR = 1.9, 95%CI = 1.5, 2.3) and thyroid cancer (SIR = 0.8, 95%CI = 0.6, 0.9); females exposed to chlamydia with other STIs, or gonorrhea with other STIs, were also 3.2‐ and 2.9‐times more likely to develop colon cancer, respectively. In males, statistically significantly associations were identified for cancer overall (SIR = 1.1, 95%CI = 1.0, 1.2) and Hodgkin lymphoma (SIR = 1.8, 95%CI = 1.0, 2.9); males exposed to chlamydia only were also 1.5‐ and 1.6‐times more likely to develop prostate and testicular cancer, respectively, while males exposed to only syphilis were 2.4‐times more likely to develop lung cancer. Our findings are consistent with common bacterial STIs being correlates of risk of certain cancers, although the possible etiologic mechanisms may be indirect.

## What's New?

While previous studies have identified the carcinogenicity of viral agents such as HIV, the role of bacterial sexually transmitted infections in carcinogenesis remains understudied despite them similarly causing chronic inflammation. This Canadian study quantified cancer risk following bacterial sexually transmitted infections with chlamydia, gonorrhea, and/or syphilis using population‐based registries. Cancer risk was increased in the infection cohort compared to the general population. Notable excesses occurred for cervical cancer among females, as well as Hodgkin lymphoma, lung cancer, and prostate cancer among males. As bacterial sexually transmitted infection rates rise, the findings highlight how these changes could impact the cancer burden.

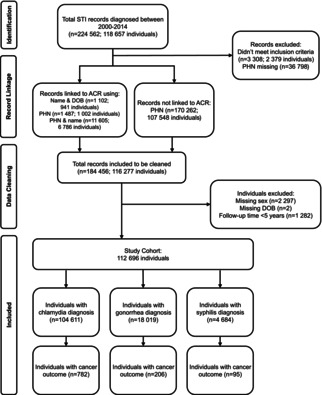

**Abbreviations**
ACR: Alberta Cancer Registry
CI: confidence interval
HIV: human immunodeficiency virus
HPV: human papillomavirus
HR: hazard ratio
ICD‐O‐3: International Classification of Diseases for Oncology, version 3
IQR: interquartile range
PHN: personal health number
SIR: standardized incidence ratio
STI: sexually transmitted infection

## 1 INTRODUCTION

Two out of every five Canadians will develop cancer during their lifetime, with an estimated 247,100 cases diagnosed in 2024 and 469,425 cases expected to be diagnosed in 2050.^1^, ^2^ As the burden of cancer is increasing due to population growth and an aging population, it is essential to identify and understand the role of new risk factors to inform cancer prevention and control initiatives given the significant burden of cancer on healthcare systems and society.^3^ One area that warrants further exploration is infectious pathogens, particularly sexually transmitted infections (STIs) given their recent rapid increases in Canada.^4^, ^5^ While previous studies identified the carcinogenicity of viral agents such as human immunodeficiency virus (HIV) and human papillomavirus (HPV), the role of bacterial STIs in carcinogenesis is less well understood despite it similarly causing chronic inflammation, a well‐established and important factor in tumorigenesis.^6^, ^7^

In Canada, chlamydia, gonorrhea, and syphilis are the most commonly reported bacterial STIs, with the incidence of these three STIs substantially increasing in the last two decades^4^, ^5^; over a 25‐year period (1998–2023), the annual rate per 100,000 increased 2‐fold for chlamydia (184.0–375.4), 6.3‐fold for gonorrhea (18.5–115.7), and 290‐fold for syphilis (0.2–58) in Alberta.^4^, ^8^ Although this is a public health concern, there remains a paucity of studies investigating whether these increases could impact other diseases, such as cancer. Indeed, few studies on this topic considered exposure to all three bacterial STIs when investigating the risk of cancer, and a comprehensive exploration of the relationship between these exposures and many cancer sites remains unexplored despite the possible systemic effect of inflammation from bacterial STIs, particularly when combined with other infectious agents. Therefore, this study aimed to determine the risk of developing cancer after exposure to chlamydia, gonorrhea, and/or syphilis in Alberta, Canada during the period of 2000–2019 using population‐based registry data.

## 2 METHODS

### 2.1 Study population

This study utilized a population‐based, retrospective cohort, record‐linkage design. A study cohort comprising all Albertan residents with at least one record of chlamydia trachomatis (henceforth referred to only as chlamydia), gonorrhea, or syphilis diagnosis from January 1, 2000 to December 31, 2014 was identified using data from STI Centralized Services at Alberta Health Services, the provincial health system for Alberta. Chlamydia, gonorrhea, and syphilis are notifiable diseases in Alberta under the Communicable Disease Regulations of the Public Health Act of Alberta^9^; thus, ascertainment is considered complete and population‐based for reported and confirmed cases. STI data included personal health number (PHN), date of birth, gender, reported STI diagnosis, diagnosis date, ethnicity, and sexual orientation at the time of STI diagnosis—all of which were recorded on the STI notification form (Data S1, Supporting Information). Since only gender was recorded on the form, biologic sex was obtained from the provincial registry, which includes all Albertan residents with an Alberta Health Care Insurance Plan, the provincial universal health care program.^10^

### 2.2 Outcome ascertainment

Using a combination of PHN, first name, last name, and date of birth, the bacterial STI cohort was linked by data custodians at Alberta Health Services to the Alberta Cancer Registry (ACR) to identify individuals diagnosed with a primary invasive cancer (excluding non‐melanoma skin cancer) or in situ bladder cancer after their first bacterial STI diagnosis. Individuals diagnosed with cancer prior to their bacterial STI diagnosis were also identified for exclusion. The ACR is legally mandated to register and collect information on all new cancer cases in the province.^11^ Cancer data included cancer registration number, diagnosis date, morphology, topography, and tumor behavior using International Classification of Diseases for Oncology, version 3 (ICD‐O‐3) codes.^12^ Cancer risk was assessed overall and for the following cancer sites where at least 20 cases were observed: brain (C71), breast (C50), lung (C34), cervix (C53), colon (C18), head and neck (C00‐14, 30‐32), Hodgkin lymphoma (C18), kidney (C64), non‐Hodgkin lymphoma (C82‐86), prostate (C61), rectum (C20), testis (C62), melanoma (C43, C69), and thyroid gland (C73). For the purposes of this study, only the first cancer diagnosis was included in the analysis because subsequent primary cancers (*n* = 67) may be related to the first cancer type or its treatment, which could bias results. Vital status and date of death, if appropriate, were obtained through linkage with the provincial Vital Statistics registry.

### 2.3 Potential risk factors

Based on prior literature of cancer risk factors, age, sex and gender, sexual orientation, and ethnicity data were assessed to identify potential differences in cancer risk within the cohort. Age at first recorded STI exposure was categorized in 20 year bands (0–19, 20–39, 40–59, 60–79, 80+). Biologic sex was assessed as a binary variable (male, female), while gender was categorized into men, women, and transgender or other, per the data collection form. Sexual orientation was aggregated into heterosexual, sexual minority, which included bisexual and same‐sex partners, and missing due to nonresponse or the individual being aged 12 years or less at the time of bacterial STI record. Finally, ethnicity was broadly categorized as either white, Indigenous, or racialized, which included all non‐Indigenous racialized groups.^13^

### 2.4 Statistical analysis

Recognizing the need to allow for lag time between the bacterial STI exposure and cancer development, follow‐up began 5 years after the date of the first bacterial STI reported during the study period and ended when the person was diagnosed with cancer, died, or the study end date (December 31, 2019), whichever occurred first. Cancer risk was assessed internally within the study cohort and externally to the general population of Alberta.

For the internal analysis, this study separately examined exposure to chlamydia, gonorrhea, and syphilis. Within each bacterial STI exposure category, the study cohort was classified into three groups: (i) “only”—those only exposed to the bacterial STI of interest, (ii) “with other STIs”—those exposed to the bacterial STI of interest and at least one other bacterial STI, and (iii) “none”—those unexposed to the bacterial STI of interest (i.e., diagnosed with one or both of the other bacterial STIs) (Table S1). To compare cancer risks between groups, crude and adjusted hazard ratios (HRs) with corresponding 95% confidence intervals (95%CIs) were computed using Cox's proportional hazard regression models for cancer overall and each site. Covariates of interest (sex, ethnicity, sexual orientation, and age at first reported STI) were incrementally added to the model based upon their significance in the crude HR model. Gender was omitted from the model due to collinearity with biologic sex as only 0.3% (*n* = 370) of the cohort had discordant sex‐gender information, only one of whom developed cancer.

For the external comparison, we explored cancer risk overall and for each cancer site within the entire cohort, as well as by each bacterial STI group for (i) those only exposed to the bacterial STI of interest and (ii) those exposed to the bacterial STI of interest and at least one other bacterial STI. Cumulative incidence probabilities for cancer overall were calculated as a function of follow‐up, with death treated as a competing risk, and were then compared to the expected cancer incidence in the general population of Alberta. Additionally, sex‐specific standardized incidence ratios (SIRs), with corresponding 95%CIs, were calculated by dividing the observed cancer count in the bacterial STI cohort by the expected cancer count in the general population; the expected number of cancers was determined by multiplying the sex‐, age‐ (5‐year bands), and calendar year‐ (1‐year band) specific cancer incidence rates for the Alberta population with the corresponding person‐years accumulated by individuals in the bacterial STI cohort.

For both internal and external comparisons, statistical significance was determined by assessing the 95%CIs, with interval ranges including 1.0 deemed nonsignificant. All statistical analyses were undertaken using Stata software, version 18.^14^

## 3 RESULTS

**FIGURE 1:**
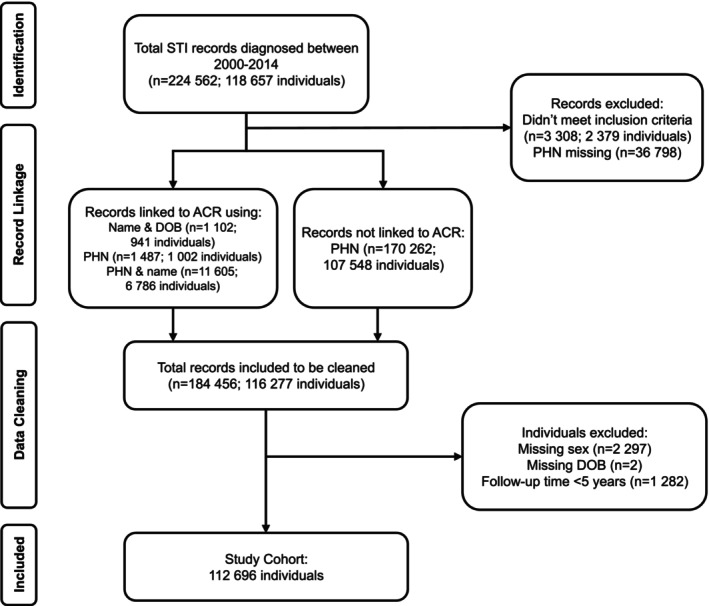
Study cohort assembly. A flow diagram depicting the study cohort assembly process, providing the number of records and individuals used or dropped at each step. ACR, Alberta Cancer Registry; DOB, date of birth; PHN, personal health number; STI, sexually transmitted infection.

A total of 112,696 individuals with 671,886 person‐years (median follow‐up = 5.5 years; IQR = 2.6, 8.9) were included in the analysis (Figure 1 and Table 1). As 92.8% of the cohort was exposed to chlamydia, these individuals influenced the demographics of the overall bacterial STI exposed cohort, which was largely female, white, and heterosexual (Table 1). Contrarily, those exposed to syphilis were more likely to be part of the racialized group (28.5% vs. 10.0% and 10.4% for chlamydia and gonorrhea, respectively), and a sexual minority (19.2% vs. 3.2% and 10.7% for chlamydia and gonorrhea, respectively). Among those exposed to gonorrhea, more than one‐third were Indigenous (38.0% vs. 15.4% and 31.4% for chlamydia and syphilis, respectively). Finally, age was not consistent across STI diagnoses as individuals exposed to syphilis were older at the time of first bacterial STI diagnosis (median age = 30 years vs. 22 years for chlamydia or gonorrhea).

**TABLE 1:** Characteristics of the study cohort.

Characteristics	Chlamydia		Gonorrhea		Syphilis		Any STI	
	Overall, *n* (%)	Individuals with cancer^a^, *n* (%)	Overall, *n* (%)	Individuals with cancer^a^, *n* (%)	Overall, *n* (%)	Individuals with cancer^a^, *n* (%)	Overall, *n* (%)	Individuals with cancer^a^, *n* (%)
Overall	104,611 (100)	782 (100)	18,019 (100)	206 (100)	4684 (100)	95 (100)	112,696 (100)	958 (100)
Sex								
Female	69,085 (66.0)	527 (67.4)	8876 (49.3)	70 (34.0)	1941 (41.4)	22 (23.2)	71,623 (63.6)	559 (58.4)
Male	35,526 (34.0)	255 (32.6)	9143 (50.7)	136 (66.0)	2743 (58.6)	73 (76.8)	41,073 (36.4)	399 (41.6)
Age at first bacterial STI diagnosis								
0–19	30,375 (29.0)	126 (16.1)	6097 (33.8)	20 (9.7)	919 (19.6)	3 (3.2)	31,214 (27.7)	128 (13.4)
20–39	69,971 (66.9)	509 (65.1)	10,386 (57.6)	90 (43.7)	2366 (50.5)	15 (15.8)	74,997 (66.5)	555 (57.9)
40–59	4120 (3.9)	135 (17.3)	1425 (7.9)	79 (38.3)	1157 (24.7)	50 (52.6)	6034 (5.4)	227 (23.7)
60–79	144 (0.1)	12 (1.5)	109 (0.6)	17 (8.3)	214 (4.6)	27 (28.4)	420 (0.4)	48 (5.0)
80+	1 (0.0)	0 (0)	2 (0.0)	0 (0)	28 (0.6)	0 (0)	31 (0.0)	0 (0)
Median (IQR)	22 (19–27)	26 (21–36)	22 (18–29)	49 (37–60)	30 (21–42)	52 (44–61)	22 (19–28)	29 (22–41)
Gender^b^								
Woman	69,240 (66.2)	526 (67.3)	8884 (49.3)	70 (34.0)	1942 (41.5)	22 (23.2)	71,773 (63.7)	558 (58.2)
Man	35,371 (33.8)	256 (32.7)	9133 (50.7)	136 (66.0)	2668 (57.0)	73 (76.8)	40,921 (36.3)	400 (41.8)
Transgender/other	0 (0)	0 (0)	2 (0.0)	0 (0)	1 (0.0)	0 (0)	2 (0.0)	0 (0)
Ethno‐racial group^b^								
White	56,734 (54.2)	448 (57.3)	6867 (38.1)	96 (46.6)	1687 (36.0)	53 (55.8)	59,817 (53.1)	534 (55.7)
Indigenous	16,093 (15.4)	85 (10.9)	6843 (38.0)	43 (20.9)	1472 (31.4)	9 (9.5)	17,582 (15.6)	98 (10.2)
Racialized^c^	10,454 (10.0)	76 (9.7)	1872 (10.4)	20 (9.7)	1336 (28.5)	26 (27.4)	12,227 (10.8)	111 (11.6)
Missing	21,330 (20.4)	173 (22.1)	2437 (13.5)	47 (22.8)	189 (4.0)	7 (7.4)	23,070 (20.5)	215 (22.4)
Sexual orientation^b^								
Heterosexual	79,138 (75.6)	527 (67.4)	13,130 (72.9)	124 (60.2)	3550 (75.8)	69 (72.6)	84,029 (74.6)	635 (66.3)
Sexual minority^d^	3378 (3.2)	29 (3.7)	1922 (10.7)	27 (13.1)	900 (19.2)	16 (16.8)	4409 (3.9)	47 (4.9)
Missing and <12 years	22,095 (21.1)	226 (28.9)	2967 (16.5)	55 (26.7)	234 (5.0)	10 (10.5)	24,258 (21.5)	276 (28.8)

Abbreviations: CI, confidence interval; ref., reference category; STI, sexually transmitted infection.

^a^ Overall cancer is all sites combined including carcinoma in situ for bladder and excluding non‐melanoma skin cancer.

^b^ As recorded in the first STI record during the study period.

^c^ Other includes Asian/black/Latin American/Middle Eastern/mixed race/non‐specified.

^d^ Sexual minority includes same‐sex and bisexual individuals.

Overall, 958 individuals (0.9%) developed cancer, with breast (*n* = 163), cervix uteri (*n* = 90), thyroid (*n* = 83), prostate (*n* = 74), and colon (*n* = 52) being the most common sites (Table S2). Compared to the overall cohort, individuals diagnosed with cancer were more likely to be male or identify as a man, older at first bacterial STI diagnosis, and White (Table 1). While these patterns were generally consistent when stratified by bacterial STI type, no differences were observed by sex or gender for chlamydia, and the proportion of sexual minorities developing cancer after gonorrhea was higher than the overall gonorrhea group (13.1% vs. 10.7%, respectively).

### 3.1 Internal comparison

The probability of a cancer diagnosis was 70% lower in individuals with a history of only chlamydia (crude HR = 0.3, 95%CI = 0.3, 0.4) compared to individuals with no chlamydia diagnosis, whereas crude HRs of 2.0 (95%CI = 1.6, 2.5) and 5.0 (95%CI = 3.9, 6.3) were observed for individuals with only gonorrhea or only syphilis, respectively, compared to their reference “none” groups (Table 2); when combined with other STIs, a decreased HR was still observed in the chlamydia with other STIs group (crude HR = 0.3, 95%CI = 0.3, 0.4), but no statistically significant differences were observed for the gonorrhea or syphilis exposure groups. After adjustment for sex, ethnicity, sexual orientation, and age at first reported bacterial STI, all statistically significant results were attenuated towards the null, notably after the inclusion of age at first reported bacterial STI, with statistically significant findings exclusively observed for the chlamydia only group (adjusted HR = 0.8, 95%CI = 0.6, 0.9) and syphilis only group (adjusted HR = 1.4, 95%CI = 1.0, 1.8), compared to their reference “none” categories.

**TABLE 2:** Hazard ratios (HR) for overall and site‐specific cancers in both sexes combined.

Cancer site	HR (95%CI)	Chlamydia exposure			Gonorrhea exposure		Syphilis exposure			
		None	Only	With other STIs	None	Only	With other STIs	None	Only	With other STIs
All	Crude HR	1 (ref)	0.3 (0.3, 0.4)	0.3 (0.3, 0.4)	1 (ref)	2.0 (1.6, 2.5)	0.9 (0.8, 1.1)	1 (ref)	5.0 (3.9, 6.3)	1.1 (0.8, 1.7)
	Adjusted HR (Model 1)^a^	1 (ref)	0.4 (0.3, 0.4)	0.3 (0.3, 0.4)	1 (ref)	1.8 (1.4, 2.2)	0.9 (0.7, 1.1)	1 (ref)	4.6 (3.6, 5.8)	1.1 (0.7, 1.7)
	Adjusted HR (Model 2)^b^	1 (ref)	0.4 (0.3, 0.4)	0.4 (0.3, 0.5)	1 (ref)	1.9 (1.5, 2.3)	1.1 (0.9, 1.3)	1 (ref)	4.7 (3.6, 6.1)	1.4 (0.9, 2.0)
	Adjusted HR (Model 3)^c^	1 (ref)	0.4 (0.3, 0.4)	0.4 (0.3, 0.5)	1 (ref)	1.8 (1.5, 2.3)	1.0 (0.8, 1.3)	1 (ref)	4.6 (3.5, 5.9)	1.3 (0.8, 1.9)
	Adjusted HR (Model 4)^d^	1 (ref)	0.8 (0.6, 0.9)	0.9 (0.7, 1.1)	1 (ref)	1.1 (0.9, 1.4)	1.1 (0.9, 1.3)	1 (ref)	1.4 (1.0, 1.8)^e^	1.0 (0.6, 1.5)
Brain	Adjusted HR (Model 4)^d^	1 (ref)	2.0 (0.4, 9.3)	2.5 (0.4, 14.3)	1 (ref)	0.4 (0.0, 2.8)	0.9 (0.2, 3.2)	1 (ref)	1.2 (0.1, 10.0)	1.2 (0.1, 10.8)
Breast	Adjusted HR (Model 4)^d^	1 (ref)	1.3 (0.6, 3.0)	1.1 (0.4, 3.0)	1 (ref)	0.9 (0.3, 2.1)	0.9 (0.5, 1.6)	1 (ref)	0.6 (0.1, 2.8)	‐
Bronchus and lung	Adjusted HR (Model 4)^d^	1 (ref)	0.6 (0.3, 1.2)	1.1 (0.4, 2.8)	1 (ref)	0.8 (0.3, 1.8)	1.4 (0.6, 3.4)	1 (ref)	2.4 (1.1, 5.1)	2.6 (0.7, 9.1)
Cervix	Adjusted HR (Model 4)^d^	1 (ref)	0.7 (0.2, 1.9)	1.1 (0.3, 3.3)	1 (ref)	1.0 (0.2, 3.9)	1.4 (0.7, 2.5)	1 (ref)	3.2 (0.8, 13.9)	1.5 (0.5, 4.9)
Colon	Adjusted HR (Model 4)^d^	1 (ref)	0.6 (0.3, 1.2)	1.4 (0.6, 3.6)	1 (ref)	1.6 (0.7, 3.8)	2.0 (0.9, 4.4)	1 (ref)	1.2 (0.4, 4.0)	0.7 (0.1, 5.6)
Head and neck	Adjusted HR (Model 4)^d^	1 (ref)	0.3 (0.1, 0.8)	0.8 (0.3, 2.5)	1 (ref)	3.5 (1.4, 8.5)	2.2 (0.8, 6.3)	1 (ref)	1.1 (0.3, 4.3)	1.0 (0.1, 8.0)
Hodgkin lymphoma	Adjusted HR (Model 4)^d^	1 (ref)	0.7 (0.2, 2.5)	0.5 (0.1, 2.7)	1 (ref)	0.9 (0.2, 4.0)	0.6 (0.1, 2.6)	1 (ref)	3.5 (0.7, 18.3)	1.5 (0.2, 12.9)
Kidney	Adjusted HR (Model 4)^d^	1 (ref)	0.5 (0.2, 1.6)	0.6 (0.1, 2.7)	1 (ref)	1.2 (0.3, 4.3)	1.0 (0.3, 3.5)	1 (ref)	2.9 (0.7, 12.1)	‐
Melanoma	Adjusted HR (Model 4)^d^	1 (ref)	4.3 (0.5, 34.2)	3.3 (0.4, 30.3)	1 (ref)	0.4 (0.0, 2.9)	0.6 (0.2, 2.0)	1 (ref)	‐	0.8 (0.1, 6.8)
Non‐Hodgkin lymphoma	Adjusted HR (Model 4)^d^	1 (ref)	0.7 (0.3, 1.9)	1.3 (0.4, 4.0)	1 (ref)	1.0 (0.3, 3.0)	1.7 (0.7, 4.1)	1 (ref)	1.8 (0.5, 6.2)	‐
Prostate gland	Adjusted HR (Model 4)^d^	1 (ref)	1.0 (0.6, 1.6)	0.7 (0.3, 1.6)	1 (ref)	1.3 (0.7, 2.6)	1.0 (0.5, 2.0)	1 (ref)	0.8 (0.4, 1.6)	1.2 (0.5, 3.2)
Rectum	Adjusted HR (Model 4)^d^	1 (ref)	0.3 (0.1, 0.9)	0.3 (0.1, 1.4)	1 (ref)	1.9 (0.6, 5.9)	0.7 (0.1, 3.1)	1 (ref)	2.4 (0.6, 9.4)	3.9 (0.8, 18.2)
Testis	Adjusted HR (Model 4)^d^	1 (ref)	‐	‐	1 (ref)	‐	0.3 (0.1, 1.3)	1 (ref)	‐	‐
Thyroid gland	Adjusted HR (Model 4)^d^	1 (ref)	0.9 (0.4, 2.2)	0.4 (0.1, 1.6)	1 (ref)	0.8 (0.2, 2.7)	0.5 (0.2, 1.4)	1 (ref)	2.3 (0.6, 7.9)	‐

*Note*: Overall cancer is all sites combined including carcinoma in situ for bladder and excluding non‐melanoma skin cancer.

Abbreviations: CI, confidence interval; HR, hazard ratio; ref., reference category; STI, sexually transmitted infection.

^a^ Model 1: Hazard ratios are adjusted for sex.

^b^ Model 2: Hazard ratios are adjusted for sex and ethno‐racial group at first reported STI.

^c^ Model 3: Hazard ratios are adjusted for sex, ethno‐racial group and sexual orientation at first reported STI.

^d^ Model 4: Hazard ratios are adjusted for sex, ethno‐racial group, sexual orientation and age at first reported STI.

^e^ Confidence limit rounded down to 1.0.

When explored by cancer types, the chlamydia only group was found to have a statistically significantly decreased HR for head and neck cancer and rectal cancer, with adjusted HRs of 0.3 observed for both, compared to those with no chlamydia exposure. Conversely, the gonorrhea only group was positively associated with head and neck cancer (adjusted HR = 3.5, 95%CI = 1.4, 8.5) compared to those with no gonorrhea diagnosis, while the syphilis only group was positively associated with lung cancer (adjusted HR = 2.4, 95%CI = 1.1, 5.1) compared to those with no syphilis diagnosis.

### 3.2 External comparison

**FIGURE 2:**
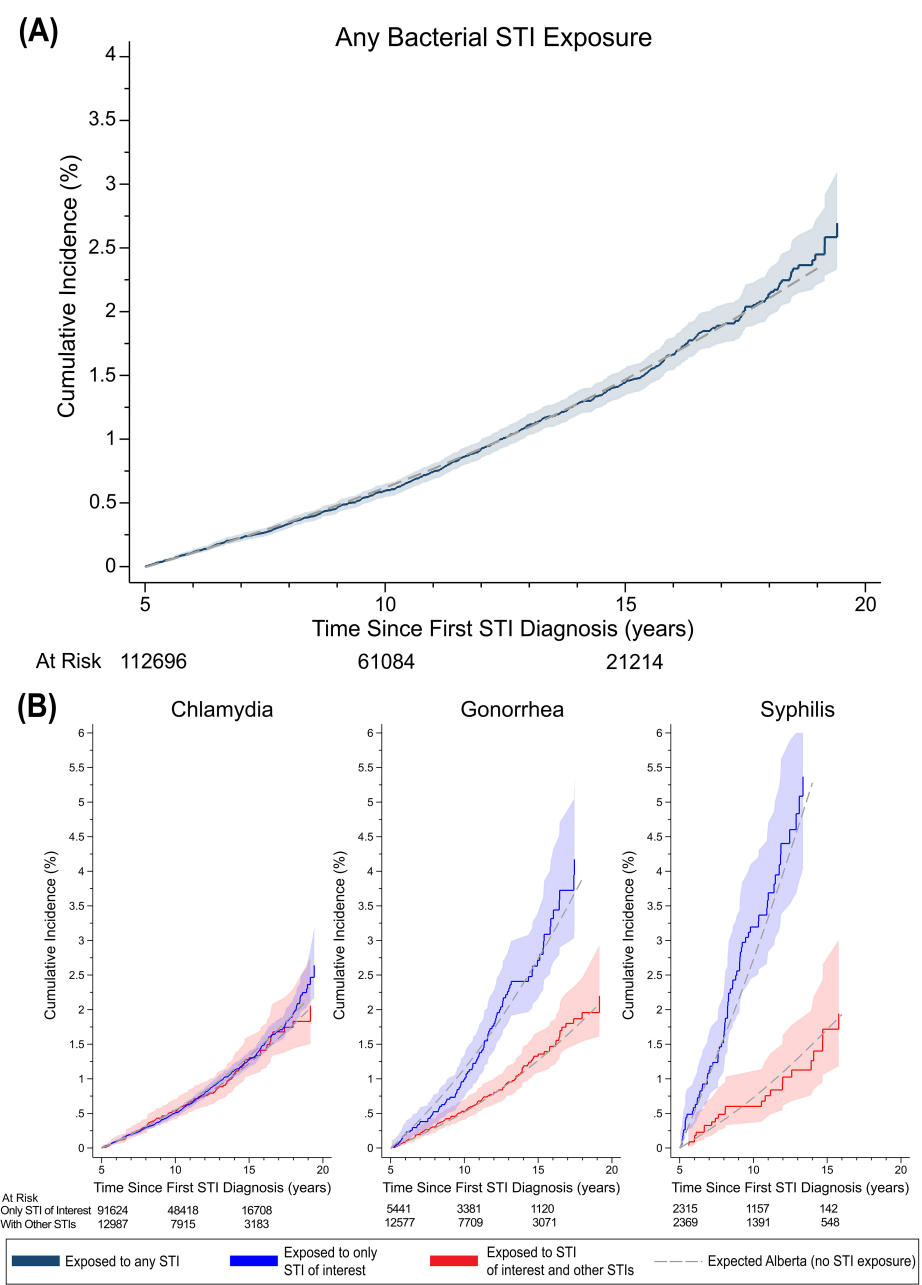
Cumulative incidence for overall cancers by STI exposure among both sexes. Cumulative incidence of overall cancer is shown as a function of time since first bacterial STI diagnosis for any STI exposure (A) and for those exposed to chlamydia, gonorrhea, and syphilis (B). For figures in (B), the cumulative incidence of overall cancer is presented in blue for those exposed to only the STI of interest and in red for those exposed to the STI of interest and at least one other bacterial STI. Expected values were derived using biological sex‐, age‐ and calendar year‐specific cancer incidence rates for Alberta. Shaded areas are 95%CIs. STI, sexually transmitted infection.

In this bacterial STI cohort, the cumulative incidence for cancer overall was 1.7% at 15 years from the first bacterial STI diagnosis (Figure 2). While cumulative incidence proportions were comparable in the chlamydia only and chlamydia with other STIs groups, at 1.4% at 15 years from the first bacterial STI diagnosis, the gonorrhea only and syphilis only groups had notably higher cumulative incidence proportions relative to the gonorrhea with other STIs and syphilis with other STIs groups, respectively (15‐year cumulative incidence: gonorrhea only = 3.3% versus gonorrhea with other STIs = 1.5%, syphilis only = 5.4% versus syphilis with other STIs = 1.9%). Importantly, all cumulative incidence proportions observed among the bacterial STI groups were comparable to the general population of Alberta.

Multiplicative excess risks for cancer among individuals with a bacterial STI exposure compared to the general population of Alberta are presented for females in Table 3 and males in Table 4. While multiplicative risks for cancer overall were not significantly elevated for females, significant associations were observed for cervical cancer (SIR = 1.9, 95%CI = 1.5, 2.3), where individuals exposed to chlamydia only (SIR = 1.7, 95%CI = 1.3, 2.1), chlamydia with other STIs (SIR = 3.4, 95%CI = 2.0, 5.4), and gonorrhea with other STIs (SIR = 1.3, 95%CI = 1.8, 5.2) were also positively associated with cervical cancer when investigated individually (Table 3). Exposure to chlamydia with other STIs and gonorrhea with other STIs was also associated with colon cancer, with SIRs of 3.2 (95%CI = 1.3, 6.7) and 2.9 (95%CI = 1.1, 6.3) observed, respectively. Conversely, a statistically significant inverse association for thyroid cancer was observed among females exposed to any bacterial STI (SIR = 0.7, 95%CI = 0.6, 0.9). Finally, there was an indication of an association with lung cancer among the chlamydia with other STIs, gonorrhea with other STIs, and syphilis with other STIs groups, though these analyses included few cancer counts. Among males with any bacterial STI diagnosis, statistically significant associations were identified for cancer overall (SIR = 1.1, 95%CI = 1.0, 1.2) and Hodgkin lymphoma (SIR = 1.8, 95%CI = 1.0, 2.9). For specific bacterial STI exposures, statistically significant associations were identified with prostate cancer (SIR = 1.5, 95%CI = 1.0, 2.1) and testicular cancer (SIR = 1.6, 95%CI = 1.1, 2.2) among the chlamydia only group, as well as lung cancer (SIR = 2.4, 95%CI = 1.2, 4.2) among the syphilis only group.

**TABLE 3:** Standardized incidence ratios (SIR) with 95% confidence intervals (CI) for females.

Cancer site	Estimates	Any STI exposure	Chlamydia exposure		Gonorrhea exposure		Syphilis exposure	
			Only	With other STIs	Only	With other STIs	Only	With other STIs
All	O/E	559/581.0	469/483.6	58/55.1	17/22.9	53/52.2	15/18.5	7/9.2
	SIR (95%CI)	1.0 (0.9, 1.0)	1.0 (0.9, 1.1)	1.1 (0.8, 1.4)	0.7 (0.4, 1.2)	1.0 (0.8, 1.3)	0.8 (0.5, 1.3)	0.8 (0.3, 1.6)
Brain	O/E	12/12.8	12/10.8	0	0	0	0	0
	SIR (95%CI)	0.9 (0.5, 1.6)	1.1 (0.6, 1.9)					
Breast	O/E	161/172.4	143/143.5	11/14.6	5/7.9	11/13.8	NR	0
	SIR (95%CI)	0.9 (0.8, 1.1)	1.0 (0.8, 1.2)	0.8 (0.4, 1.3)	0.6 (0.2, 1.5)	0.8 (0.4, 1.4)	0.3 (0.0, 1.2)	
Bronchus and lung	O/E	16/15.2	10/11.1	NR	NR	NR	NR	NR
	SIR (95%CI)	1.1 (0.6, 1.7)	0.9 (0.4, 1.7)	3.7 (1.0, 9.4)^a^	0.9 (0.0, 4.9)	3.9 (1.1, 9.9)	0.6 (0.0, 3.1)	4.6 (0.1, 25.7)
Cervix	O/E	90/48.0	69/41.1	17/5.0	NR	15/4.8	NR	NR
	SIR (95%CI)	1.9 (1.5, 2.3)	1.7 (1.3, 2.1)	3.4 (2.0, 5.4)	1.6 (0.2, 5.8)	3.1 (1.8, 5.2)	3.8 (0.5, 13.8)	3.9 (0.8, 11.3)
Colon	O/E	22/23.2	12/18.9	7/2.2	NR	6/2.1	NR	NR
	SIR (95%CI)	0.9 (0.6, 1.4)	0.6 (0.3, 1.1)	3.2 (1.3, 6.7)	1.0 (0.0, 5.7)	2.9 (1.1, 6.3)	1.8 (0.2, 6.4)	2.7 (0.1, 15.2)
Head and neck	O/E	12/9.8	10/8.2	0	NR	0	0	0
	SIR (95%CI)	1.2 (0.6, 2.1)	1.2 (0.6, 2.2)		5.2 (0.6, 18.8)			
Hodgkin lymphoma	O/E	8/13.6	7/11.5	0	0	0	NR	0
	SIR (95%CI)	0.6 (0.3, 1.2)	0.6 (0.2, 1.3)				13.2 (0.3, 73.8)	
Kidney	O/E	12/11.8	11/9.8	NR	0	NR	0	0
	SIR (95%CI)	1.0 (0.5, 1.8)	1.1 (0.6, 2.0)	1.0 (0.0, 5.3)		1.0 (0.0, 5.6)		
Melanoma	O/E	35/43.3	33/36.6	NR	0	NR	0	NR
	SIR (95%CI)	0.8 (0.6, 1.1)	0.9 (0.6, 1.3)	0.5 (0.1, 1.7)		0.2 (0.0, 1.4)		1.4 (0.0, 8.0)
Non‐Hodgkin lymphoma	O/E	20/18.6	18/15.4	NR	0	NR	0	0
	SIR (95%CI)	1.1 (0.7, 1.7)	1.2 (0.7, 1.8)	1.1 (0.1, 4.0)		1.2 (0.1, 4.2)		
Rectum	O/E	10/10.2	7/8.4	NR	0	NR	NR	0
	SIR (95%CI)	1.0 (0.5, 1.8)	0.8 (0.3, 1.7)	1.1 (0.0, 6.4)		1.2 (0.0, 6.7)	4.9 (0.6, 17.5)	
Thyroid gland	O/E	69/92.2	62/78.6	NR	NR	NR	NR	0
	SIR (95%CI)	0.7 (0.6, 0.9)	0.8 (0.6, 1.0)	0.4 (0.1, 1.0)	0.8 (0.1, 2.9)	0.4 (0.1, 1.1)	0.9 (0.0, 5.0)	

Abbreviations: E, expected cancers; NR, not reportable with observed cancer count between 1 and 4; O, observed cancers.

^a^ Confidence limit rounded up to 1.0.

**TABLE 4:** Standardized incidence ratios (SIR) with 95% confidence intervals (CI) for males.

Cancer site	Estimates	Any STI exposure	Chlamydia exposure		Gonorrhea exposure		Syphilis exposure	
			Only	With other STIs	Only	With other STIs	Only	With other STIs
All	O/E	399/354.0	205/184.6	50/47.1	79/68.4	57/50.1	56/47.6	17/16.3
	SIR (95%CI)	1.1 (1.0, 1.2)	1.1 (1.0, 1.3)^a^	1.1 (0.8, 1.4)	1.2 (0.9, 1.4)	1.1 (0.9, 1.5)	1.2 (0.9, 1.5)	1.0 (0.6, 1.7)
Brain	O/E	15/10.6	9/6.7	NR	NR	NR	NR	NR
	SIR (95%CI)	1.4 (0.8, 2.3)	1.3 (0.6, 2.6)	2.7 (0.7, 6.9)	0.7 (0.0, 3.7)	2.0 (0.4, 5.8)	1.3 (0.0, 7.5)	2.6 (0.1, 14.2)
Bronchus and lung	O/E	31/22.1	10/8.2	NR	6/5.6	NR	12/5.0	NR
	SIR (95%CI)	1.4 (1.0, 2.0)^a^	1.2 (0.6, 2.3)	0.7 (0.1, 2.6)	1.1 (0.4, 2.3)	1.0 (0.2, 2.8)	2.4 (1.2, 4.2)	1.6 (0.2, 5.7)
Colon	O/E	30/25.0	18/13.0	NR	7/4.8	NR	NR	0
	SIR (95%CI)	1.2 (0.8, 1.7)	1.4 (0.8, 2.2)	0.9 (0.2, 2.7)	1.5 (0.6, 3.0)	0.9 (0.2, 2.5)	0.6 (0.1, 2.1)	
Head and neck	O/E	21/16.4	6/8.4	5/2.2	7/3.4	5/2.3	NR	NR
	SIR (95%CI)	1.3 (0.8, 2.0)	0.7 (0.3, 1.6)	2.3 (0.7, 5.4)	2.1 (0.8, 4.3)	2.2 (0.7, 5.1)	1.4 (0.3, 4.0)	1.3 (0.0, 7.0)
Hodgkin lymphoma	O/E	16/8.8	11/6.3	NR	NR	NR	NR	NR
	SIR (95%CI)	1.8 (1.0, 2.9)	1.7 (0.9, 3.1)	1.5 (0.2, 5.5)	2.3 (0.3, 8.3)	1.5 (0.2, 5.6)	4.2 (0.1, 23.4)	3.8 (0.1, 21.3)
Kidney	O/E	16/18.3	8/10.0	NR	NR	NR	NR	0
	SIR (95%CI)	0.9 (0.5, 1.4)	0.8 (0.3, 1.6)	0.8 (0.1, 3.0)	0.9 (0.2, 2.5)	0.8 (0.1, 2.8)	1.4 (0.3, 4.0)	
Melanoma	O/E	12/21.9	9/12.7	NR	NR	NR	0	0
	SIR (95%CI)	0.5 (0.3, 1.0)^a^	0.7 (0.3, 1.3)	0.7 (0.1, 2.5)	0.3 (0.0, 1.5)	0.7 (0.1, 2.4)		
Non‐Hodgkin lymphoma	O/E	21/20.9	8/12.1	5/2.8	NR	5/2.9	NR	0
	SIR (95%CI)	1.0 (0.6, 1.5)	0.7 (0.3, 1.3)	1.8 (0.6, 4.1)	1.1 (0.3, 2.9)	1.7 (0.6, 4.0)	1.9 (0.5, 4.7)	
Prostate gland	O/E	74/60.0	34/22.9	7/7.7	19/15.5	10/8.6	11/12.4	5/3.5
	SIR (95%CI)	1.2 (1.0, 1.5)^a^	1.5 (1.0, 2.1)	0.9 (0.4, 1.9)	1.2 (0.7, 1.9)	1.2 (0.6, 2.1)	0.9 (0.4, 1.6)	1.4 (0.5, 3.3)
Rectum	O/E	12/14.1	NR	NR	5/2.9	NR	NR	NR
	SIR (95%CI)	0.8 (0.4, 1.5)	0.6 (0.2, 1.4)	0.5 (0.0, 3.0)	1.7 (0.6, 4.0)	0.5 (0.0, 2.8)	0.5 (0.0, 2.9)	3.0 (0.4, 10.7)
Testis	O/E	37/30.2	35/22.4	NR	0	NR	0	0
	SIR (95%CI)	1.2 (0.9, 1.7)	1.6 (1.1, 2.2)	0.4 (0.1, 1.6)		0.5 (0.1, 1.6)		
Thyroid gland	O/E	14/14.5	11/9.6	0	NR	0	NR	0
	SIR (95%CI)	1.0 (0.5, 1.6)	1.1 (0.6, 2.0)		0.5 (0.0, 2.9)		2.5 (0.3, 9.1)	

Abbreviations: E, expected cancers; NR, not reportable with observed cancer count between 1 and 4; O, observed cancers.

^a^ Confidence limit rounded up to 1.0.

## 4 DISCUSSION

To our knowledge, this is the first study in Canada to explore the relationship between bacterial STIs and cancer. By linking data from two population‐based health registries over a 20‐year period, we assembled a large, contemporary cohort to investigate this unique exposure‐outcome relationship using three bacterial STI exposures, which were assessed individually and in combination with other bacterial STI exposures. Although our internal analyses suggested the likelihood of developing cancer was largely comparable within the bacterial STI cohort, head and neck cancer was more common among those exposed exclusively to gonorrhea, and there was an indication that cancer overall, as well as particularly lung cancer, was more common among those exposed only to syphilis, even after adjustment. Additionally, in our external comparisons to the general population of Alberta, we identified statistically significantly elevated incidence of cancer overall in males; statistically significant associations were also identified for specific cancer types, namely cervical and colon cancer in females and lung, prostate, and testicular cancer, as well as Hodgkin lymphoma, in males. Contrarily, we found a significant inverse association of thyroid cancer among females exposed to bacterial STIs. These findings add to the literature regarding the possible relationship between bacterial STIs and cancer, and identify new exposure‐cancer associations that warrant further investigation.

The most consistent finding of our study was the statistically significant association of cervical cancer after bacterial STI exposure. These findings correspond with previous literature, including a meta‐analysis published in 2016 that reported a two‐fold increased odds of cervical cancer after chlamydia exposure (OR = 2.2, 95%CI = 1.9, 2.6, *p* = <.001),^15^ and multiple studies that identified prior gonorrhea infection significantly increased the risk of cervical cancer.^16^, ^17^ However, a limitation of our study is our inability to disentangle the impact of the three bacterial STIs investigated from HPV exposure.^18^ HPV infection is a necessary cause of invasive cervical cancer,^19^ though there is evidence that chlamydia and gonorrhea infections further increase the risk of cervical cancer in females with HPV, with these bacterial STIs acting as possible HPV cofactors in its etiology.^15^, ^20^ Further investigation of cervical cancer and HPV risk with bacterial STI exposure data is evidently needed to understand the independent roles of chlamydia and gonorrhea in cervical cancer development.

Another notable association identified in this study is the statistically significant association between only syphilis exposure and lung cancer, which was found in our internal comparison and external comparison among males. To date, there is no evidence of a biological mechanism for lung cancer development after syphilis. However, it is possible that due to the longevity, severity, and systemic nature of syphilis infection, there is an increased risk of carcinogenesis.^21^ HIV exposure may also act as a mediator, as previous literature identified an increased prevalence of HIV among those with a syphilis diagnosis compared to the general population and those exposed to chlamydia and gonorrhea,^22^, ^23^ and lung cancer is reported to be 2‐ to 7‐times higher in HIV‐infected individuals compared to the general population.^24^ While HIV status was largely unknown for this cohort, there was an indication that HIV‐positive status was more prevalent in the syphilis‐exposed group, regardless of cancer outcome (data not shown). Differential smoking behaviors among individuals previously diagnosed with syphilis compared to those exposed to chlamydia or gonorrhea, as well as the general population, may also confound the observed association with lung cancer.^25^ Similarly, those exposed to syphilis were more likely to be classified as a sexual minority, with previous studies suggesting this population experiences elevated cancer risk factors including HIV, smoking, and alcohol consumption compared to the heterosexual population.^5^, ^26^ Given the rate of syphilis is rising faster than other bacterial STIs,^4^, ^8^ further research is needed to understand cancer risks in this population with increased statistical power.

Other novel findings of this study were the associations between chlamydia‐only exposure and testicular and prostate cancer. While the literature surrounding infections and testicular cancer is limited, a recent review reports that HIV and Epstein–Barr virus can increase risk, but evidence for an association with bacterial STIs is conflicting.^27^ Indeed, a previous case–control study even contradicts our findings, suggesting gonorrhea exposure increased the odds of testicular cancer by 93% (95%CI = 1.02–3.63) compared to controls.^28^ Similarly, a recent scoping review of STIs and prostate cancer, which included 33 studies investigating the effect of chlamydia, did not support an association.^29^ Given the conflicting findings in the literature, the role of bacterial STIs in the development of these male sex‐specific cancers should be an area of investigation for future studies.

Our internal comparison also suggested a positive association between gonorrhea‐only exposure and head and neck cancer, with our external comparison identifying SIRs of 2.1 and 2.2 among males with gonorrhea‐only exposure or gonorrhea with other STIs exposure, respectively, though these did not reach statistical significance. This finding corresponds with a recent, nationwide Danish case–control study^30^ and may be related to more risky oral sexual behaviors among individuals with a past gonorrhea exposure. Specifically, pharyngeal gonorrhea is more prevalent than urogenital gonorrhea,^31^, ^32^ and may cause inflammation in the head and neck region independently or through increased oral HPV infection.^33^ However, other important risk factors for head and neck cancer are tobacco smoking and alcohol consumption, both of which are more common in the bacterial STI exposed population compared to the general population,^26^, ^34^, ^35^, ^36^ and thus these findings remain to be confirmed with more robust studies.

To our knowledge, this is also the first study to identify an association between bacterial STIs and colon cancer, with a 3‐fold higher risk observed in females exposed to chlamydia with other STIs or gonorrhea with other STIs, compared to the general population. While the prior literature has focused more extensively on anal cancers, particularly among males,^37^, ^38^, ^39^ due to HPV infection being its most important risk factor,^39^ recent studies have similarly identified an increased risk of colorectal cancer with HPV, especially HPV 16 and 18 infection.^40^ Given the paucity of studies on this topic, further research is needed to rule out the possibility of a Type I error.

Interestingly, we found that females with a past bacterial STI exposure had a significantly lower incidence of thyroid cancer compared to the general population. We hypothesize this inverse association does not reflect a protective effect, but rather a lack of overdetection of thyroid cancer in this population.^41^ This may be because populations that are underserved by the Canadian healthcare system were overrepresented in our bacterial STI cohort, including sexual minorities and racialized groups.^42^ Consequently, it is possible that the female bacterial STI population is interacting with the healthcare system at a lower capacity than the general population or is receiving less investigative care (e.g., full body exams, medical imaging), which has resulted in a decreased likelihood of overdetecting indolent thyroid cancer.

Finally, due to the inclusion of socioeconomic factors and sexual behaviors on the bacterial STI notification form, we were able to comment on notable variation in cohort characteristics between the specific bacterial STI exposure groups and contextualize our findings relative to the general population of Alberta. For example, while those exposed to chlamydia were younger and appeared to be the most similar to the overall Albertan population, those exposed to gonorrhea and syphilis were more likely to identify with a key, underserved population and were older at first bacterial STI diagnosis, the latter of which resulted in higher cumulative incidence proportions for cancer given it is a disease of aging. Similarly, the study cohort had an elevated proportion of Indigenous peoples compared to the Alberta population at the time (15.6% vs. 5.8%–6.5%),^43^ with more than one‐third of those exposed to gonorrhea identified as Indigenous. Further, while the cohort was less likely to belong to a racialized group compared to the general population (10.8% vs. 13.9%–23.5%),^44^ we observed that those exposed to syphilis were more than twice as likely to be classified as racialized than those exposed to gonorrhea or chlamydia.^5^ Finally, it was estimated in 2018 that 4% of Canadians identified as non‐heterosexual,^45^ which is markedly lower than the prevalence of sexual minorities in the gonorrhea (10.7%) and syphilis (19.2%) exposed groups. These identity characteristics interact with the risk of both bacterial STI exposure and cancer, potentially impacting the associations observed in this study.^5^, ^26^, ^46^, ^47^ Nonetheless, through this initial exploration, we highlight the need for intersectionality‐based research in this topic as it is critical to informing targeted public health measures.^5^

The clear strengths of our study include its population‐based nature and strong underlying data sources that are mandated to collect bacterial STI and cancer data with high levels of completeness. The study design also allowed minimal chance of selection bias as the cancer status did not influence the selection of bacterial STI exposed individuals. In addition, the population‐based design and large cohort size based on 15 years of data made it possible to separately explore three bacterial STI exposures and their association with multiple cancer sites. Despite these strengths, we must acknowledge several limitations. First, while our investigation of ethnicity, gender, and sexual orientation is meritorious given the paucity of literature including such variables, misclassification is possible given the uncertainty as to whether the variables are self‐reported by the patient or reported by the administrator, as well as the fact that gender and sexual orientation can be fluid. Further, while adjustment of these factors in the internal comparisons was feasible, our external comparison could not account for these underlying differences in our bacterial STI cohort compared to the general population of Alberta as ethnicity, race, and sexual orientation are not recorded during cancer registration. Second, the study period was selected as STI data were incomplete before 2000, resulting in possible misclassification of individuals born before 2000 as these individuals could have had a bacterial STI diagnosis prior to our study start date. Thirdly, chlamydia and gonorrhea have been shown to resolve spontaneously and thus underreporting of these bacterial STIs is possible.^48^ Fourth, there is a potential for Type I errors due to the number of statistical associations explored. Finally, the most significant limitation of our study was our inability to examine the effects of possible confounders for some cancer associations, such as tobacco^26^, ^34^, ^35^, ^49^ and alcohol use,^36^, ^49^ HIV, and HPV. Although we did receive data on HIV status, this information was largely incomplete (unknown HIV status = 82.9%), and only a few cancers were observed in HIV‐positive individuals (*n* = 14), preventing stratification by this variable. In the case of HPV, it is not a reportable disease in Alberta and is highly transient in nature^5^; as Alberta started an HPV vaccination program among girls of school age in 2008, which was later extended to boys,^50^ it will be possible to control for possible confounding effects of HPV by investigating cancer risk in an HPV‐vaccinated population in the future.

In conclusion, this study provides the first Canadian exploration of the association between common bacterial STIs and cancer, providing an in‐depth investigation using both internal and external comparisons. In addition to novel associations, our findings indicate that the risk of cancer is not uniform for bacterial STI exposures and that many extraneous factors may be impacting cancer risk in this population. We have thus highlighted the need for future research to elucidate the role of bacterial STIs in cancer development, particularly when the effects of known cancer risk factors are appropriately measured. This research is imperative to public health measures as the culture surrounding sex changes and infection rates of bacterial STIs continue to rise.

## AUTHOR CONTRIBUTIONS

**Hina M. Qureshi:** Writing – original draft; formal analysis; conceptualization; data curation; methodology; visualization; writing – review and editing. **Taylor Hughes:** Formal analysis; writing – review and editing; data curation; visualization; methodology. **Eduardo L. Franco:** Writing – review and editing; methodology. **Kirsten M. Fiest:** Writing – review and editing; methodology. **Jennifer Gratrix:** Writing – review and editing; methodology. **Petra A. Smyczek:** Writing – review and editing; methodology. **Ronald Read:** Writing – review and editing; methodology. **Arfan R. Afzal:** Writing – review and editing; methodology. **Rob Deardon:** Writing – review and editing; methodology. **Aliya Kassam:** Writing – review and editing; methodology. **Miranda M. Fidler‐Benaoudia:** Writing – review and editing; conceptualization; methodology; supervision.

## CONFLICT OF INTEREST STATEMENT

The authors declare no conflicts of interest.

## ETHICS STATEMENT

Ethical approval was obtained from the Health Research Ethics Board of the Alberta‐Cancer Committee (HREBA.CC‐20‐0092).

## Supporting information


**Data S1.** Alberta Health Services notification of sexually transmitted infection form.
**Table S1.** Bacterial STI‐specific matric of proportions among STI exposed population.
**Table S2.** Cancer sites and observed cancer counts in both sexes combined.

## Data Availability

Further information and anonymized aggregate data are available from the corresponding author upon request.
