# Supplementary material for: Risk of cancer among individuals with a history of bacterial sexually transmitted infections: A population‐based study in Alberta, Canada
Source: Int J Cancer. 2025 Nov 14;158(5):1383–95. doi: 10.1002/ijc.70215 (PMC12765966; doi:10.1002/ijc.70215)
Supplement: Supplementary file 1 — Data S1. Alberta Health Services notification of sexually transmitted infection form. Table S1. Bacterial STI‐specific matric of proportions among STI exposed population. Table S2. Cancer sites and observed cancer counts in both sexes combined. [file IJC-158-1383-s001.pdf]

Risk of cancer among individuals with a history of bacterial sexually transmitted infections: a population-based study in Alberta, Canada

Hina M. Qureshi, Taylor Hughes, Eduardo L. Franco, Kirsten M. Fiest, Jennifer Gratrix, Petra A. Smyczek, Ronald Read, Arfan R. Afzal, Rob Deardon, Aliya Kassam, Miranda M. Fidler-Benaoudia

Supplementary Material

Table of Contents

Supplementary Material 1. Alberta Health Services Notification of Sexually Transmitted Infection form 2

Supplementary Table 1. Bacterial STI-specific matric of proportions among STI exposed population 3

Supplementary Table 2. Cancer sites and observed cancer counts in both sexes combined ... 4

Supplementary Material 1. Alberta Health Services Notification of Sexually Transmitted Infection form

Alberta

Notification of Sexually Transmitted Infections

This form is to be completed and returned within 2 weeks of lab notification by a Healthcare Professional.

- **The *Public Health Act* requires that all Sexually Transmitted Infections cases and their contacts be reported to STI Centralized Services.**
- **The Partner Notification Nurse (PNN) may contact your patient to obtain additional information.**
- **For questions regarding the treatment of a case or ordering of medications call 780-735-1466 or toll free 1-888-535-1466.**

Section 1 - Personal Identifiers (Please PRINT)

Patient Name *Last Name* *First Name* *Middle Name*
Gender ☐ Male ☐ Female ☐ X ☐ Transgender

Current Address ☐ **Homeless** Municipality Province and Country

Postal Code | Telephone Number | Birth Date (yyyy-mm-dd) | Personal Health Number | Marital Status

Country of Birth | Arrival date in Canada (yyyy-mm-dd) | Place of Work
Pregnant ☐ Yes ➔ *If Yes, Test of Cure (TOC) recommended (see page 2)* ☐ No ☐ EDD

Lives on Reserve? ☐ Yes ☐ No ☐ Unknown ➔ *If Yes, name of First Nations Community*
Post natal (<=6 weeks) ☐ Yes ➔ *Delivery Date* ☐ No

Ethnic Group ☐ Caucasian ☐ African/Caribbean/Black ☐ Asian (East/SE) ☐ Other Asian ☐ Middle East/Arab ☐ Latin American ☐ First Nations ☐ Inuit ☐ Métis ☐ Unknown ☐ Other *(specify)*

Current Social / Behavioural History (X all that apply)
☐ People who injects drugs ☐ Sex with males ☐ Sex with females
☐ Sex with People who injects drugs ☐ Worker: Exchange Goods/Money for Sex
☐ Anonymous Partner ☐ Patron: Exchange Goods/Money for Sex

Reason for visit (X all that apply)
☐ STI Screening ☐ Symptoms ☐ Contact ☐ Pre/Postnatal
☐ Sexual Assault ☐ Therapeutic Abortion ☐ Other *(specify)*

Section 2 - Clinical Findings

Clinical Findings (X all that apply) ☐ Not Examined — Duration

				Duration
Symptomatic	☐ Yes	☐ No	☐ Unknown	
⌘ Dysuria	☐ Yes	☐ No	☐ Unknown	
Rectal Symptoms	☐ Yes	☐ No	☐ Unknown	
Sores / Lesion / Rash	☐ Yes	☐ No	☐ Unknown	
Abn Vaginal Discharge	☐ Yes	☐ No	☐ Unknown	
★ Cervical Discharge	☐ Yes	☐ No	☐ Unknown	N/A
★ Friable Cervix	☐ Yes	☐ No	☐ Unknown	N/A
⌘ Urethral Discharge	☐ Yes	☐ No	☐ Unknown	
Other	☐ Yes	☐ No	*If Yes, specify:*	

Complications (X all that apply)
☐ PID ☐ Epididymo-Orchitis ☐ Other *(specify)*

Blood Tests Ordered
Syphilis ☐ Yes ☐ No HIV ☐ Yes ☐ No

Drug Allergy
☐ Yes ☐ No *If Yes, specify:*

Section 3 - Treatment Details (X all that apply)

Notifiable Infections	Code*	Patient Treated With
☐ Chlamydia Trachomatis	A	☐ Azithromycin (1 g)
☐ Gonorrhea	B	☐ Cefixime (800 mg)
☐ Non-Gonococcal Urethritis ⌘	C	☐ Amoxicillin (500 mg tid x 7 days)
☐ Mucopurulent Cervicitis ★	D	☐ Azithromycin (2 g)
☐ Syphilis	E	☐ Ceftriaxone (250 mg IM)
☐ Chancroid	F	☐ Doxycycline (100 mg bid x 7 days)
☐ Lymphogranuloma Venereum	G	☐ Doxycycline (100 mg bid x 14 days)
	H	☐ Doxycycline (100 mg bid x 28 days)
Date of Treatment (yyyy-mm-dd)	I	☐ Levofloxacin (500 mg qd x 14 days)
	J	☐ Metronidazole (500 mg bid x 14 days)
Provider's Name (please PRINT)	K	☐ Special Drugs *(see reverse)*
	L	☐ Other *(specify name and dosage)*
		☐ Not treated

Mailing Address:
➔ Mailing Address is required to ensure drug replacement *(see reverse for more information).*

USE OFFICE STAMP OR MAILING LABEL.

- **Please send a separate notification form if additional contacts are identified.**

Section 4 - Sexual Contact One Information

Contact Name *Last* *First* *Middle*
Gender ☐ Male ☐ Female ☐ X ☐ Transgender | Birthdate (yyyy-mm-dd) | Age | Marital Status
Current Address
Municipality | Province and Country | Postal Code
Telephone Number | Cell Number
Occupation and Place of Work
Distinguishing Features | Ethnicity *(see Section 1)*
Date and Location of Exposure
Relationship to Patient ☐ Current Regular Partner ☐ Casual Known ☐ Ex-Regular Partner ☐ Anonymous Partner ☐ Exchange Goods/Money for Sex
Provider treated Contact? ☐ Yes ☐ No Date (yyyy-mm-dd) Medication Code* *(see Section 3)*

Section 5 - Sexual Contact Two Information

Contact Name *Last* *First* *Middle*
Gender ☐ Male ☐ Female ☐ X ☐ Transgender | Birthdate (yyyy-mm-dd) | Age | Marital Status
Current Address
Municipality | Province and Country | Postal Code
Telephone Number | Cell Number
Occupation and Place of Work
Distinguishing Features | Ethnicity *(see Section 1)*
Date and Location of Exposure
Relationship to Patient ☐ Current Regular Partner ☐ Casual Known ☐ Ex-Regular Partner ☐ Anonymous Partner ☐ Exchange Goods/Money for Sex
Provider treated Contact? ☐ Yes ☐ No Date (yyyy-mm-dd) Medication Code* *(see Section 3)*

Mail all copies, sealed in the envelope provided to: STI Services, SSP South Tower, 200-10030 107 Street NW, Edmonton, Alberta, T5J 3E4.

AH0332 Rev. 2019-06 **Indicate if you require any of the following:** ☐ Billing Invoice Attached ☐ Notification Forms ☐ Patient Literature

Supplementary Table 1. Bacterial STI-specific matrix of proportions among STI exposed population

	Chlamydia only	Gonorrhea only	Syphilis only	Chlamydia and gonorrhea	Chlamydia and syphilis	Gonorrhea and syphilis	Chlamydia, gonorrhea, and syphilis	Total
	n(%)	n(%)	n(%)	n(%)	n(%)	n(%)	n(%)	n
**Chlamydia**								
None	0 (0.0)	5441 (67.3)	2315 (28.6)	0 (0.0)	0 (0.0)	329 (4.1)	0 (0.0)	8085
Only	91624 (100.0)	0 (0.0)	0 (0.0)	0 (0.0)	0 (0.0)	0 (0.0)	0 (0.0)	91624
With other STIs	0 (0.0)	0 (0.0)	0 (0.0)	10947 (84.3)	738 (5.7)	0 (0.0)	1302 (10.0)	12987
**Gonorrhea**								
None	91624 (96.8)	0 (0.0)	2315 (2.4)	0 (0.0)	738 (0.8)	0 (0.0)	0 (0.0)	94,677
Only	0 (0.0)	5441 (100.0)	0 (0.0)	0 (0.0)	0 (0.0)	0 (0.0)	0 (0.0)	5441
With other STIs	0 (0.0)	0 (0.0)	0 (0.0)	10947 (87.0)	0 (0.0)	329 (2.6)	1302 (10.4)	12578
**Syphilis**								
None	91624 (84.8)	5441 (5.0)	0 (0.0)	10947 (10.1)	0 (0.0)	0 (0.0)	0 (0.0)	108,012
Only	0 (0.0)	0 (0.0)	2315 (100.0)	0 (0.0)	0 (0.0)	0 (0.0)	0 (0.0)	2315
With other STIs	0 (0.0)	0 (0.0)	0 (0.0)	0 (0.0)	738 (31.2)	329 (13.9)	1302 (55.0)	2369
**Total Study Cohort**	91624 (81.3)	5441 (4.8)	2315 (2.1)	10947 (9.7)	738 (0.7)	329 (0.3)	1302 (1.2)	112696

Supplementary Table 2. Cancer sites and observed cancer counts in both sexes combined[a]

Cancer site (ICD-O-3)	Cancer count
Bladder (c67)	19
Bone and connective tissue (c40-41, c49)	16
Brain (c27)	27
Breast (c50)	163
Bronchus and lung (c34)	47
Cervix uteri (c53)	90
Colon (c18)	52
Endometrium (c41)	17
Head and Neck (c00-14, c30-32)	33
Hodgkins Lymphoma (965-966)	24
Kidney (c64)	28
Leukemia (980-994)	19
Liver & intrahepatic bile ducts (c22)	14
Melanoma (c44, c69, 872-879)	47
Multiple myeloma and plasmacytoma	9
Non-Hodgkins Lymphoma (c41, 956, 967-972)	41
Other hematopoietic and reticuloendothelial (974,975, 995-999)	17
Ovary (c56)	18
Pancreas (c25)	8
Prostate gland (c61)	74
Rectum (c20)	22
Stomach (c16)	14
Testis (c62)	37
Thyroid gland (c73)	83
Other cancer sites[b] (c21, c24, c26, c15, c57, c23, c38, c76, c75, c55, c540, c542-c549, c60, c19, c48, c17, c80, c66, c51)	39
**Total**	**958**

[a] Includes only first subsequent cancers for all three STI exposures combined. Cancers counts were not separately presented for male, and female due to the presence of non-reportable (1-5) cancer count in some cells.
[b] Cancer sites having an overall count equal to or less than 5. This category includes anus anal canal, biliary tract other unspecified, digestive organs other & ill-defined, esophagus, female genital organs other & unspecified, gall bladder, heart mediastinum & pleura, other & ill-defined sites, other endocrine glands and related structures, other uterus & uterus NOS, penis, rectosigmoid junction, retroperitoneum and peritoneum, small intestine, unknown primary, ureter, vulva
